# Quasi-real-time photon pulse duration measurement by analysis of FEL radiation spectra

**DOI:** 10.1107/S1600577515022997

**Published:** 2016-01-01

**Authors:** Robin Engel, Stefan Düsterer, Günter Brenner, Ulrich Teubner

**Affiliations:** a Deutsches Elektronen-Synchrotron, Notkestrasse 85, D-22603 Hamburg, Germany; b Institut für Physik, Carl von Ossietzky Universität Oldenburg, D-26111 Oldenburg, Germany; c Institut für Laser und Optik, Hochschule Emden/Leer, University of Applied Sciences, Constantiaplatz 4, D-26723 Emden, Germany

**Keywords:** FLASH, free-electron laser, SASE, photon pulse, photon pulse duration, spectral correlation analysis

## Abstract

For photon diagnostics at free-electron lasers (FELs), the determination of the photon pulse duration is an important challenge and a complex task. This is especially true for SASE FELs with strongly fluctuating pulse parameters. However, most techniques require an extensive experimental setup, data acquisition and evaluation time, limiting the usability in all-day operation. In contrast, the presented work uses an existing approach based on the analysis of statistical properties of measured SASE FEL spectra and implements it as a software tool, integrated in FLASH’s data acquisition system. This allows the calculation of the average pulse durations from a set of measured spectral distributions with only seconds of delay, whenever high-resolution spectra are recorded.

## Abstract 2

Considering the second-order spectral correlation function of SASE-FEL radiation allows a real-time observation of the photon pulse duration during spectra acquisition.

## 1. Introduction   

Fourth-generation extreme ultraviolet (XUV) or X-ray light sources such as free-electron lasers (FELs) provide ultra-fast pulse durations ranging from a few to hundreds of femto­seconds, a spectral peak brightness which exceeds those of third-generation light sources like storage-ring-based synchrotrons or high-order harmonics of intense laser femtosecond pulses (Teubner & Gibbon, 2009 ▸) by several orders of magnitude, and a high degree of transverse spatial coherence. Such intense and ultra-short photon pulses allow for unprecedented experiments in various fields of research, *e.g.* studying atomic/molecular dynamics at ultra-fast time scales or exploring nonlinear phenomena in light–matter interactions. For experiments of this type and for the correct interpretation of the obtained data, the precise knowledge of the pulse duration is essential. Furthermore, many experiments require even a control of these ultra-short pulses during run time. However, at FELs, like the XUV/soft X-ray laser FLASH in Hamburg (Ackermann *et al.*, 2007 ▸), that are based on the self-amplified spontaneous emission (SASE) process, the laser beam properties exhibit strong pulse-to-pulse fluctuations that make measurements of the temporal pulse structure tremendously challenging. The development of an appropriate and routinely available pulse duration measurement method has therefore seen intense efforts, resulting in a variety of direct and indirect diagnostic techniques (see Düsterer *et al.*, 2014 ▸; Helml *et al.*, 2014 ▸, and references therein). Most of these schemes, however, require complex experimental setups and significant set-up times. Furthermore, in many cases a lengthy and sophisticated evaluation process follows the data taking, considerably limiting the usability of most of the diagnostic methods for setting up, tuning (and controlling) the FEL to a desired pulse duration. In general, an online diagnostics providing single-shot or averaged photon pulse duration in real time (or with only few seconds latency) combined with a minimum of set-up time is required.

In this paper we present the implementation of a quasi-real-time photon pulse duration diagnostics at FLASH through the analysis of the statistical properties of measured SASE FEL spectral distributions. Since SASE FEL radiation spectra exhibit poor overall temporal coherence and the phase of the electric field is lost during the acquisition, a direct Fourier transform to obtain the temporal XUV photon pulse profile from the intensity distribution in the frequency domain is not possible. However, the width of the spectral spikes (often denoted as longitudinal modes) can be interpreted as spectral coherence which yields a relation to the photon pulse duration (Krinsky & Gluckstern, 2003 ▸), as will be shown in the following section.

## 2. Method   

A SASE FEL operating in the linear regime can be considered as a narrowband amplifier which selectively amplifies density fluctuations resulting from shot noise of the electron beam current. The radiation spectrum emitted by the SASE process is calculated as 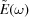 = 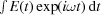 with 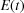 being the FEL electric field. Furthermore, spectral coherence can be expressed by the integral over the first-order correlation function (also called the ‘field correlation function’) of a SASE radiation spectrum (Saldin *et al.*, 1998 ▸). However, the first-order correlation function of the electric field cannot be measured directly. Considering the second-order correlation function (also called the ‘intensity correlation function’), which is connected to the first-order correlation function by the Siegert relation (Saldin *et al.*, 1998 ▸) and defined as
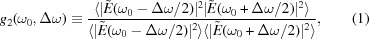
where 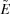 is the spectral electric field strength and 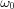 is the central FEL frequency, it becomes evident that 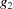 can in principle be determined by measuring the spectral intensity distribution of a photon pulse.

**Figure 1:**
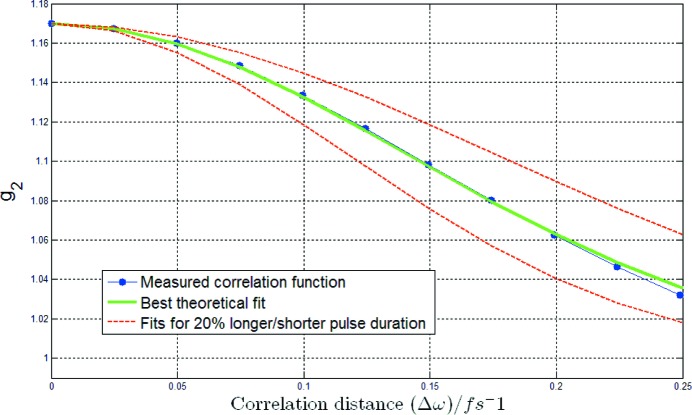
Measured second-order correlation function (blue) and fitted theoretical estimate are shown for Gaussian (green) electron bunch form. The red lines represent theoretical estimates for pulses with a varied pulse duration by ±20%.

Saldin *et al.* (1998 ▸) have shown that for the SASE process this second-order correlation function can be approximated by
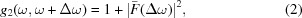
where the form factor 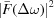 represents the squared absolute value of the Fourier-transformed temporal electron bunch profile 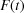. For FLASH, the longitudinal electron bunch profile can be considered as good approximation to be of Gaussian shape (Behrens *et al.*, 2012 ▸; Düsterer *et al.*, 2014 ▸). Thus the second-order correlation function can be rewritten as a function of the electron bunch duration 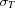:
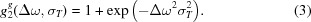



In general, the spectrometer output signal 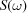 for a single-shot spectrum can be written as the spectral intensity distribution
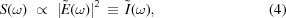
assuming a sufficiently good spectrometer resolution. With the assumption that the temporal structure of the electron bunch is retained during the amplification process (linear amplification) and the spectrometer resolution is much narrower than the FEL gain bandwith, it can be shown that the spectral intensity correlation function can be computed from recorded spectra as (Lutman *et al.*, 2012 ▸)
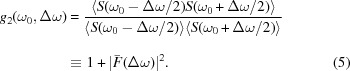
By fitting the experimental 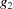 function with the analytical model, the photon pulse duration can be derived (see also Fig. 1 ▸). It should be noted that here at FLASH the plane-grating monochromator (PG) beamline used in spectrometer mode (see below) provides a resolving power that is much higher than needed for this approach. The treatment for a limited spectrometer resolution is described by Lutman *et al.* (2012 ▸).

The second-order correlation method is proposed for electron bunches with a uniform electron energy. However, real electron bunches usually exhibit a temporal chirp of the electron energy within the bunch. This affects the spectral coherence. Effectively, the second-order correlation method measures the degree of spectral coherence 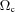 in order to retrieve the pulse duration. If 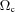 is altered by an energy chirp, the results of the second-order correlation method will be affected accordingly.

A way to compensate for this effect has been proposed by Serkez (2012 ▸). The energy chirp is considered by a linear ‘chirp-correction’ factor to the photon pulse duration 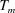 calculated using the second-order correlation method, thus retrieving a corrected photon pulse duration 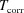:
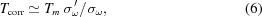
where 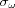 is the FEL gain bandwidth and 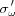 the spectral bandwidth of the FEL radiation measured by the PG spectrometer. The FEL gain bandwidth 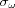 depends on the bandwidth of the electron kinetic energy distribution which in principle can be measured using a transverse deflecting RF-structure (TDS) integrated in the FEL accelerator section (Röhrs *et al.*, 2009 ▸). It should be noted that the correction factor is often large (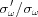 ≃ 2–3) and only accommodates linear chirp. The correct measurement of this correction factor is therefore one of the major limits to the absolute precision of the pulse duration measurement.

## 3. Instrumentation   

This section briefly describes the employed experimental setup and developed software tools. All FEL radiation spectra that have been evaluated in the studies presented here were recorded at the permanently installed high-resolution PG beamline at FLASH. The PG beamline was operated in spectrometer mode, delivering highly resolved single-shot spectra at a repetition rate of 10 Hz and a resolving power of 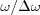 ≃ 10000. A full review of the beamline can be found by Martins *et al.* (2006 ▸) and Gerasimova *et al.* (2011 ▸). It should also be noted that the acquisition of spectra at the PG beamline is an invasive measurement which cannot be performed in parallel to other experiments. However, any online spectrometer with sufficient resolution like, for example, the variable-line-spacing grating spectrometer installed at the BL beamline branch at FLASH (Brenner *et al.*, 2011 ▸) can be used for spectral recording.

As mentioned before, for the complete analysis the electron bunch properties, and in particular the energy chirp, have to be measured in addition. The longitudinal phase space of an electron bunch is recorded by a TDS, installed downstream of the FLASH accelerator section (Altenmueller *et al.*, 1964 ▸; Röhrs *et al*., 2009 ▸; Behrens *et al*., 2012 ▸). The TDS projects the longitudinal bunch shape into the vertical transverse plane. This streaked profile is recorded on a screen by a camera. In combination with a dipole magnet dispersing the bunch perpendicular to the streak direction the TDS allows a direct single-shot longitudinal phase space measurement of the electron bunch. By doing so the energy chirp correction factor can be determined and incorporated in the photon pulse duration evaluation. Note that during these measurements no parallel SASE operation is possible. Fortunately, the electron beam properties do not change significantly on a shot-to-shot basis. Thus, a reference measurement of the electron bunch shape and potential chirp is needed only once for a given accelerator setting.

The above-described analysis method was implemented into the FLASH data acquisition system (Engel, 2015 ▸). This way, any recorded spectra from the PG beamline can be evaluated to yield the photon pulse duration. The analysis software accumulates sets of spectra of variable size (of the order of 100 spectra per set). The second-order correlation function is subsequently calculated for each set and the respective photon pulse duration is computed. In addition, the resulting electron gain bandwidth 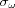 as determined by the TDS measurements can be included for electron energy chirp correction. The resulting photon pulse duration is averaged over the last measured sets and displayed in the FLASH control system.

## 4. Results and discussion   

**Figure 2:**
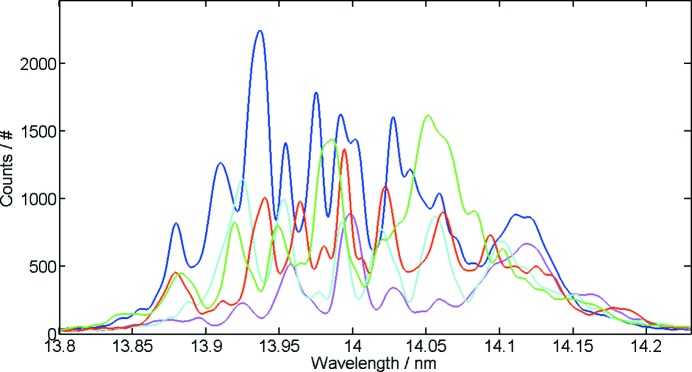
A set of typical FLASH SASE spectra recorded at the PG beamline for a central wavelength of 14 nm. The colored lines represent single-shot spectral distributions showing the spiky nature of the radiation spectrum. The statistical analysis of the spike distribution yields a good estimation of the temporal photon pulse duration. For these spectra an average pulse duration of 72 fs (FWHM) was determined.

A set of typical FLASH spectra recorded at the PG beamline is shown in Fig. 2 ▸. The random shot-to-shot fluctuations and the spiky nature of the SASE radiation become evident. The analyzed spectra are typically taken with FLASH operating at the onset of saturation regime. For typical FLASH parameters this corresponds to a few tens of µJ pulse energy.

The analytical considerations for this method are originally derived for the linear mode of SASE FEL operation. However, the analysis of numerically simulated data sets has confirmed the applicability also for the nonlinear regime (Lutman *et al.*, 2012 ▸). In a previous study it was validated that the method is in good agreement with a whole set of different pulse duration measurement techniques for typical FLASH parameters (Düsterer *et al.*, 2014 ▸). The spectral analysis scheme used by Düsterer *et al.* (2014 ▸) utilizes the same methodology as described here. Other approaches compiled in the article by Düsterer *et al.* mostly tackled the problem of XUV pulse duration determination in a much more direct way, thus serving as a good reference measurement. However, the majority of the diagnostic techniques used sophisticated experimental setups, and their measurement and analysis time was much longer, resulting in only a few measured pulse durations within several hours.

**Figure 3:**
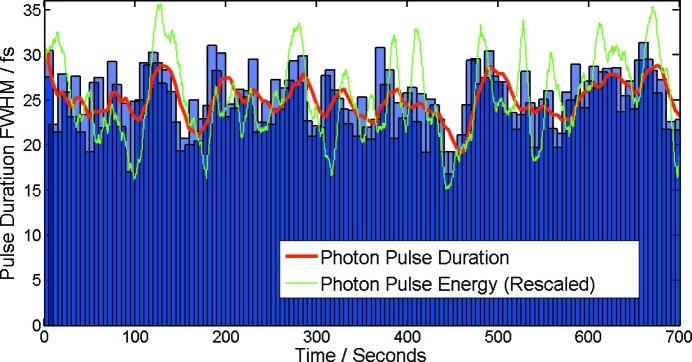
Comparison of the pulse durations derived by the second-order correlation and the averaged photon pulse energy. The spectral correlation algorithm considered sets of each 100 spectra with an overlap of 50 spectra. Each blue bar represents one such set, the red line their running average. For comparison, the green line shows the slow fluctuation of the photon pulse energy. Experimental parameters: radiation wavelength, 20.9 nm; electron bunch charge, 0.18 nC.

In contrast, the spectral analysis has the potential to deliver a value for the pulse duration every few seconds and thus the temporal evolution of pulse duration fluctuations can be followed on this time scale. In Fig. 3 ▸ the fluctuations of measured pulse durations recorded over a 12 min time period can be seen. In the plot each bar corresponds to an average pulse duration evaluated by the correlation analysis of 100 spectra recorded within 10 s acquisition time. The average pulse duration (each bar corresponds to a 10 s average value) shows fluctuations within tens of seconds where the pulse duration varies over a range from 20 fs to 30 fs (FWHM). Due to the averaging of each data point (bar) in Fig. 3 ▸ the fluctuations are not attributed to the inherent statistical shot-to-shot variations expected from the SASE process. The changes are rather attributed to slight parameter changes in the accelerator. For example, small variations of electron beam properties may influence the lasing part of the electron bunch (see, for example, Düsterer *et al.*, 2014 ▸). Hence the pulse duration variation is expected to be correlated with the overall number of created photons, *i.e.* the pulse energy. Indeed, the independently measured pulse energies, over the same time period (Tiedtke *et al.*, 2008 ▸, 2009 ▸), show a notable trend to fluctuate in unison with the pulse duration data. Up to now only a few data sets have been collected, but all show a certain degree of positive linear correlation between pulse duration and pulse energy. For the detailed data analysis and correct interpretation of many FLASH experiments this information might be of critical relevance.

The fast response of the second-order correlation analysis method also allows exploration of the influence of different FLASH machine conditions on the statistical properties of the measured spectra and thus on the photon pulse duration. In the previously mentioned extensive experimental campaign performed by Düsterer *et al.* (2014 ▸) the measurements still took a considerable amount of time, such that only two distinctly different machine settings were tested. In contrast to that, the prompt response of the spectral analysis applied here allows in principle a much larger number of parameters, *e.g.* peak currents, electron bunch charge *etc.*, to be investigated.

One question that was investigated in several experimental campaigns (Behrens *et al.*, 2012 ▸; Düsterer *et al.*, 2014 ▸) was that of the lasing fraction of the electron bunch. Due to the nonlinear interaction of the lasing process the lasing part of the electron pulse is typically only 33–66% of the electron bunch duration. The exact ratio of the two pulse durations, however, strongly depends on the accelerator setting.

**Figure 4:**
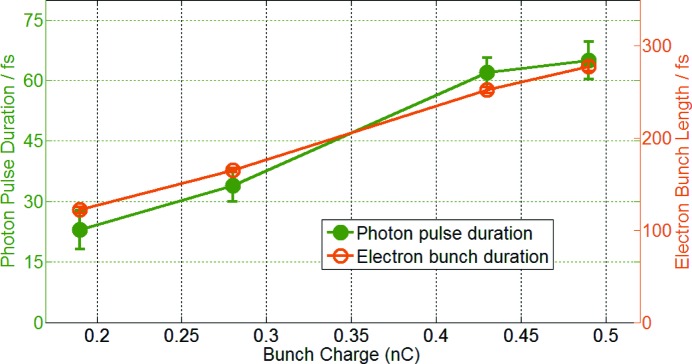
Comparison between measured photon pulse duration and electron bunch length for different bunch charges. The photon pulse duration was calculated using the second-order correlation spectral intensity analysis. The electron bunch length was measured by the TDS. The error bars of the photon pulse duration measurement represent the intrinsic standard deviation of the second-order correlation results.

For more insight the electron bunch duration was changed from 120 fs to 280 fs (FWHM) by altering the electron bunch charge over the range 0.2–0.5 nC, while leaving other machine parameters constant. The electron bunch duration was measured using the TDS as described above. The corresponding photon pulse durations calculated by the second-order correlation analysis were significantly shorter than values measured so far (23%). However, recently published results from another SASE FEL (Makita *et al.*, 2015 ▸) show similar ratios of electron bunch and photon pulse bandwidth, indicating comparable lasing fractions (26–43%). As shown in Fig. 4 ▸, the relative increase of the electron and photon pulse duration is almost constant, implying that the lasing fraction of the electron bunch stays constant for the same pulse shape but different lengths. In order to have an even better understanding of the correlation both quantitatively and qualitatively, further systematic investigations have to be performed in future.

## 5. Conclusion and outlook   

The second-order correlation analysis of XUV spectra (Lutman *et al.*, 2012 ▸; Inubushi *et al*., 2012 ▸; Düsterer *et al.*, 2014 ▸) was implemented as a real-time tool at FLASH providing average XUV pulse duration measurements with only few seconds delay to the data acquisition. This way, the photon pulse duration can be observed by both the operators tuning the accelerator and the users performing the experiments. Furthermore, the results are recorded in the data acquisition system of FLASH and are available for later analysis by the user groups. The ability to monitor the pulse duration changes online and to compare the result with other pulse parameters (*e.g.* the photon pulse energy) was demonstrated. We have also shown the monitoring of changes in the photon pulse duration while accelerator parameters are changed (*e.g.* bunch charge). The presented method provides a valuable tool for future investigations of the dependency of different machine operation settings on the pulse duration. While the method works well, there might be limits: in particular, it is unclear how far this parameter range can be extended. Defining these boundary conditions in which the spectral correlation analysis method works reliably and where the assumptions begin to fail is the subject of future work.
